# The functions of immune system-derived miRNAs in cardiovascular diseases

**DOI:** 10.1016/j.ncrna.2024.11.004

**Published:** 2024-11-14

**Authors:** Albert Sufianov, Murad Agaverdiev, Andrey Mashkin, Tatiana Ilyasova

**Affiliations:** a Educational and Scientific Institute of Neurosurgery, Рeoples’ Friendship University of Russia (RUDN University), Moscow, Russia; b Department of Neurosurgery, Sechenov First Moscow State Medical University (Sechenov University), Moscow, Russia; c Bashkir State Medical University, Ufa, Republic of Bashkortostan, 3 Lenin Street, 450008, Russia

**Keywords:** Cardiovascular diseases, miRNAs, Immuno-miRs, Immune system cells, Therapy, Personalized medicine

## Abstract

Cardiovascular diseases (CVD) are the foremost cause of mortality worldwide, with recent advances in immunology underscoring the critical roles of immune cells in their onset and progression. MicroRNAs (miRNAs), particularly those derived from the immune system, have emerged as vital regulators of cellular functions within the cardiovascular landscape. This review focuses on "immuno-miRs," a class of miRNAs that are highly expressed in immune cells, including T cells, B cells, NK cells, neutrophils, and monocytes/macrophages, and their significant role in controlling immune signaling pathways. Highlighting recent studies in human and animal models, this review examines how miRNAs influence both innate and adaptive immune responses and explores their potential as therapeutic targets for CVD. Special emphasis is placed on miRNAs that regulate T cells, suggesting that targeted manipulation of these miRNA pathways could offer new strategies for CVD treatment. As research in cardiovascular immunology advances, this review aims to provide a thorough overview of the potential of immune system-derived miRNAs to revolutionize CVD management and therapy, addressing a major global health challenge.

## 1 Introduction

Currently, cardiovascular diseases (CVDs) are the leading cause of morbidity and mortality in the world [1]. Its impact is twofold, imposing a significant direct financial burden on healthcare systems while simultaneously diminishing productivity through the loss of workdays due to illness. The heart and vasculature, at the core of CVD, con-sist of cell types that do not originate in hematopoietic tissues which include cardiomyocytes, fibroblasts, endothelial cells (ECs), vascular smooth muscle cells (VSMCs)/smooth muscle cells (SMCs), pericytes, and stromal cells [2,3]. However, in addition to this structural ensemble, there is a recognized presence of immune cells within cardiovascular tissues. These immune cells comprise various major subtypes, such as lymphocytes and myeloid-derived populations. Certain entities take residence in cardiovascular tissues, while others infiltrate these tissues. They play diverse roles, influencing alterations in both the structural and functional aspects of the heart and vasculature. This dual influence has the potential to exacerbate the continuous advancement of CVDs, highlighting the urgent necessity for comprehending and tackling CVDs. Effectively addressing the intricate interaction between the immune system and the cardiovascular system is pivotal in addressing this worldwide health challenge.

**Fig. 1:**
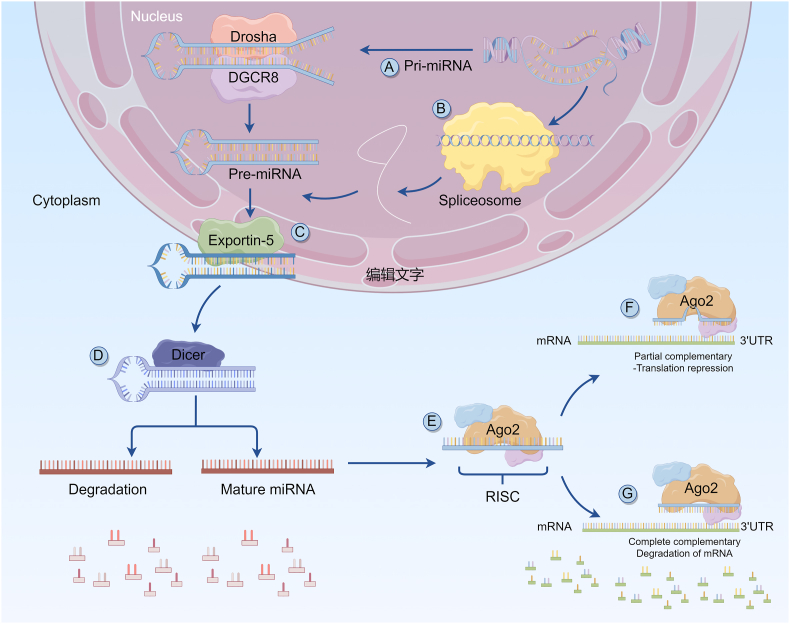
Biogenesis of microRNA (miRNA).

In the past two decades, a growing realization has dawned on us regarding the profound contributions of immune cells to the vast field of cardiovascular biology. These roles span a wide landscape, from their vital involvement in the intricate processes of heart development to their vigilant guardianship of vascular homeostasis and their participation in a complex web of physiological functions. Immune cells have emerged as sentinels, standing guard against the insidious encroachments of dis-ease-related pathologies. Understanding and addressing immune responses that either underpin or exacerbate specific diseases requires us to grasp the fundamental architects of immune cell activity and function. MicroRNAs (miRNAs) are endogenously expressed RNA molecules of 18–22 nucleotides in length that repress gene expression at the posttranscriptional level by binding to the 3′ - untranslated region (3′-UTR) of target messenger RNAs (mRNAs) (Fig. 1). MiRNAs have been shown to play an essential role in various biological processes, including the cell cycle, apoptosis, cell proliferation and differentiation.

**Fig. 2:**
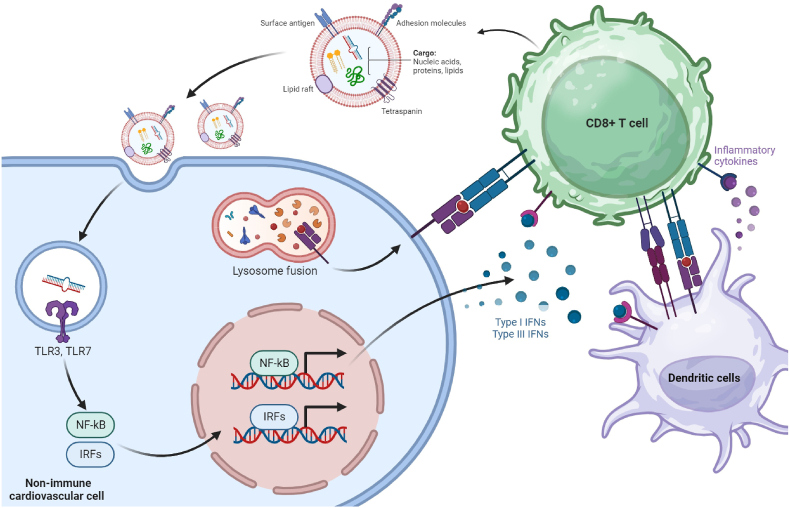
Depicts a schematic representation of cellular communication between cardiovascular and immune cells facilitated by extracellular vesicles (EVs). Under pathological conditions, immune cells can initiate an inflammatory process in cardiovascular system cells by modulating specific microRNAs (miRNAs) and target genes.

MiRNAs, often likened to orchestrators of cellular activities, hold a central role as crucial regulators of immune cell functionality and identity [4]. This comprehensive review, marked by steadfast dedication, aims to be a source of knowledge, shedding light on the extensive literature surrounding miRNAs originating from immune cells. It meticulously analyzes their roles in coordinating the intricate symphony of immune responses within the broader context of CVDs and their interconnected risk factors. This exploration into the realm of miRNAs goes beyond immune cells; it extends its reach to uncover potential roles played by miRNAs derived from the immune system. These miRNAs act as molecular messengers transmitted to non-immune cardiovascular cell types through the intricate network of extracellular vesicles (EVs) (Fig. 2). As the symphony builds to its climax, the review concludes with an examination of miRNA regulation within regulatory T cells (Tregs), proposing that finely tuning miRNA pathways within these cells may unlock a promising therapeutic avenue in the realm of Tregs immunotherapy for CVDs.

Recent advances have been made in identifying specific miRNAs involved in cardiovascular disease mechanisms, revealing their potential for diagnostic and therapeutic applications [5]. This study focuses on findings from the past five years (2018–2022), providing insights from experimental studies in human samples that illustrate the growing knowledge in this area. To collect comprehensive and relevant studies, the researchers conducted a systematic search using databases such as SCOPUS and Web of Science using keywords such as “miRNA or microRNA,” “cardiovascular disease,” “myocardial infarction,” “heart injury,” and “heart failure.” This rigorous search yielded 59 articles that were carefully assessed to ensure their relevance and scientific contribution to understanding miRNA functions in cardiovascular disease. The results of the selected studies highlight that although miRNAs serve as potent gene regulators, the detailed mechanisms by which they exert their effects remain only partially understood. This gap highlights the ongoing need for up-to-date data to elucidate the pathways that miRNAs participate in at the cellular level, particularly in relation to cardiovascular disease. Such insights could be invaluable not only in advancing our understanding of cardiovascular pathology, but also in identifying miRNAs as novel diagnostic and therapeutic (theranostic) tools, collectively known as “TheranoMIRNA”. As the field matures, the discovery of TheranoMIRNA holds great promise soon, particularly in providing personalized targeted interventions for patients with cardiovascular disease. However, there remains a pressing need for more rigorously designed studies before these molecules become widely accepted in clinical practice. These studies will be essential to provide additional evidence, validate the findings, and refine our understanding of miRNA pathways in cardiovascular disease.

## 2 MiRNAs in immune cell development and function

**Fig. 3:**
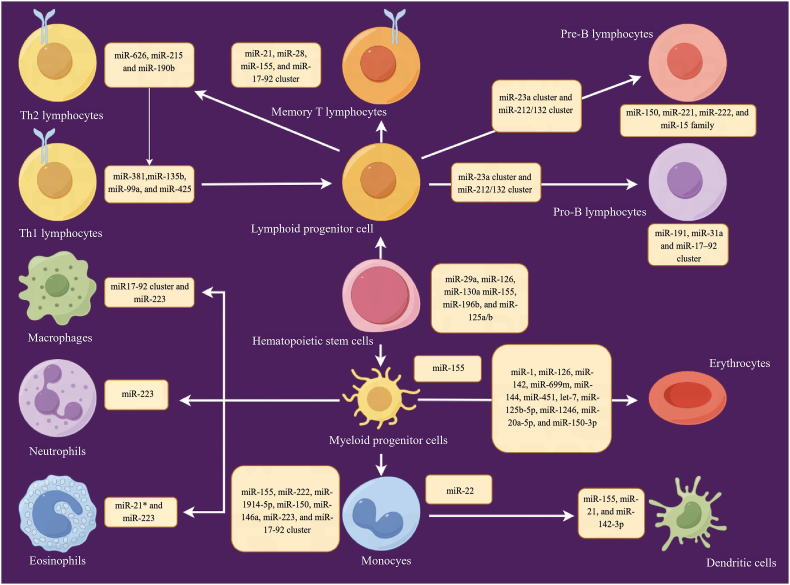
Provides a summary of the engagement of certain microRNAs (miRNAs) in the formation of diverse immune cells. Findings indicate that both the miR-17-92 cluster and miR-223 impact the differentiation of mammalian immune cells, influencing the results of immune responses to infections, and contributing to the onset of diseases with immunological origins.

MiRNAs are pivotal regulators in the complex system that govern the development, regulation, and activation of the immune system [6]. While the traditional pathways responsible for miRNA generation are well-documented, recent advancements in research have illuminated unconventional routes to miRNA biogenesis and introduced innovative mechanisms that govern these small yet powerful molecules. These discoveries carry profound implications, given the extensive documentation of miRNA expression profile irregularities across various disease contexts. These contexts encompass not only CVDs and autoimmune disorders but also the intricate landscape of tumorigenesis. Such recognition underscores the potential of miRNAs as invaluable tools for disease detection and essential instruments for meticulous monitoring of a wide range of ailments [7,8]. Within the intricate realm of immune system cells, specific miRNAs, aptly referred to as "immuno-miRs," take center stage due to their conspicuous presence and exceptional roles in shaping and fine-tuning immune responses (Fig. 3).

This subset of miRNAs forms a dynamic and ever-expanding ensemble as re-searchers continuously unearth new members, each contributing to the rich tapestry of immune system regulation. Yet, amid this diverse array of immuno-miRs, a select few rise to prominence, warranting an in-depth exploration of their profound significance [8]. These exceptional immuno-miRs not only offer invaluable insights into the intricate inner workings of the immune system but also hold the potential to become pivotal components in therapeutic interventions and diagnostic methodologies. Therefore, a comprehensive understanding of their functions and the regulatory mechanisms governing them is crucial for advancing our comprehension of the dynamic nature of the immune system and its far-reaching implications in a multitude of disease conditions. With the ongoing evolution of the field of miRNA research, further revelations are expected to continue unveiling the pivotal roles that these molecular entities play in directing the harmonious symphony of the immune system.

The immune system's role in CVDs is increasingly recognized as pivotal, with immune responses significantly influencing disease development and progression. Chronic inflammation, mediated by both innate and adaptive immune cells, is a central mechanism in CVD pathogenesis. This inflammatory environment is perpetuated by interactions between immune cells and cardiovascular tissue, which leads to tissue remodeling, fibrosis, and eventual loss of function. Immune-derived miRNAs further modulate these interactions, influencing immune responses at a molecular level and offering potential as biomarkers and therapeutic targets. Macrophages, as primary mediators of inflammation, are integral to both the initiation and resolution of immune responses in CVD. Activated macrophages adopt distinct phenotypes—pro-inflammatory (M1) or anti-inflammatory (M2)—depending on environmental cues. M1 macrophages produce inflammatory cytokines such as TNF-α and IL-6, which drive the progression of atherosclerotic plaques and contribute to myocardial damage post-myocardial infarction (MI). In contrast, M2 macrophages play a reparative role, resolving inflammation and promoting tissue repair. However, in CVD, this balance is often skewed toward M1 polarization, leading to chronic inflammation and adverse cardiac remodeling. CD4^+^ T helper cells are critical in CVD, particularly in atherosclerosis and hypertension. T-helper 1 (Th1) cells, which produce interferon-gamma (IFN-γ), drive atherosclerotic plaque formation by promoting macrophage activation and plaque instability. Regulatory T cells (Tregs), which usually counterbalance inflammatory responses, are often functionally impaired in CVD, leading to unregulated inflammation and disease progression. In addition, CD8^+^ cytotoxic T cells contribute to endothelial damage in hypertension, further highlighting the multifaceted role of T cells in CVD. While less prominent than macrophages or T cells, B cells contribute to CVD through antibody production and cytokine secretion. B cells are implicated in the pathogenesis of atherosclerosis through the production of autoantibodies that target oxidized low-density lipoprotein (oxLDL), a component of atherosclerotic plaques. These autoantibodies can exacerbate inflammation, further promoting plaque development. Neutrophils, as first responders to sites of inflammation, are highly active in CVD pathology. They release reactive oxygen species (ROS) and neutrophil extracellular traps (NETs), which amplify local inflammation and contribute to thrombus formation, a common precursor to myocardial infarction. NETs trap platelets and red blood cells, promoting the coagulation cascade and leading to vascular occlusion. Elevated neutrophil activity and NET formation are associated with increased risk of acute cardiovascular events.

In this context, specific miRNAs assume a central role in shaping the innate immune response, with their dysregulation contributing to various inflammatory diseases. Among these miRNAs, miR-223 stands out as highly expressed and tightly regulated in hematopoietic cells. It serves as a critical modulator for myeloid cell differentiation and activation [9]. Extensive research has unveiled the central role of miR-223 in modulating myeloid cell behavior, particularly in the differentiation and activation of neutrophils and macrophages. MiR-223 promotes granulopoiesis while inhibiting macrophage differentiation, delicately balancing the myeloid cell repertoire. This finely tuned control is of utmost importance, as uncontrolled myeloid activation can have severe consequences in the context of inflammatory diseases. MiR-223 acts as a vital negative feedback mechanism to restrain excessive innate immune responses, ensuring the maintenance of myeloid cell homeostasis [9]. Its role as a guardian against unchecked inflammation positions it as a vital player in preserving immune balance. Some research demonstrated a deep dive into the impact of miR-223 within the context of inflammatory diseases, with a particular focus on conditions such as acute respiratory distress syndrome and inflammatory bowel disease [9]. Was showed how miR-223 influences disease progression and delves into its potential therapeutic applications, shedding light on its promise as a target for intervention in these conditions. Beyond its roles in myeloid cells, this review also considers the non-myeloid functions of miR-223, highlighting its diverse contributions to various biological processes and pathways. The potential therapeutic applications of miR-223 take center stage in this review. Harnessing miR-223 to mitigate inflammatory responses and restore immune balance holds promise as a treatment strategy for controlling excessive innate immune reactions during mucosal inflammation. MiR-223's multifaceted functions in myeloid cells and its pivotal role in modulating innate immune responses establish it as a significant player in both immunity and inflammatory diseases. As our understanding of miR-223's diverse roles continues to expand, its therapeutic potential emerges as a beacon of hope for managing inflammatory conditions and restoring immune equilibrium during mucosal inflammation.

The miR-17/92 cluster, situated within the domain of miRNA clusters, has garnered substantial attention as one of the most extensively researched and well-documented clusters [10]. Since its initial discovery, this cluster, along with its individual members, has been the subject of consistent and exponential interest within the scientific community. A remarkable milestone was reached in 2012 when over 1000 articles were published on this cluster alone. While its primary recognition was associated with its role in tumorigenesis, recent research initiatives have unveiled a complex tapestry of unforeseen functions performed by the constituents of this cluster, spanning a wide range of biological contexts. The significance of the miR-17/92 cluster has now expanded beyond cancer and encompasses diverse settings, including normal development, immune disorders, cardiovascular diseases, neurodegenerative conditions, and the intricate process of aging. This evolution in our understanding highlights the cluster's ever-increasing importance and its continuously growing roles as a regulator of fundamental biological processes. This comprehensive review embarks on a journey to delve into the latest body of knowledge concerning the miR-17/92 cluster, with a primary emphasis on its involvement in both health and disease. Beyond a mere recapitulation of its established roles, this review aspires to offer a fresh perspective on the extensive array of protein-coding and non-coding transcripts that are likely intricately regulated by the cluster's individual members. The miR-17/92 cluster, having transcended its origins in tumorigenesis, has now emerged as a pivotal player in CVDs via immune system dysfunction [10].

MiRNAs have emerged as critical regulators in orchestrating T cell development, activation, and differentiation, with miR-181a holding a central role in this regulatory network [11]. MiR-181a′s capacity to modulate multiple signaling pathways places it as a vital controller of T cell receptor (TCR) activation thresholds, both during thymic selection and peripheral T cell responses. With advancing age, a decline in miR-181a ex-pression, attributed to reduced transcription of pri-miR-181a, leads to T cell activation defects. The exploration commences with a thorough investigation into the intricate transcriptional regulation of miR-181a. The authors identify a potential enhancer for pri-miR-181a, situated at chromosome 1 position 198,904,300 [11]. This enhancer falls under the governance of a transcription factor complex that includes Yin Yang 1 (YY1). Comprehending the transcriptional regulation of miR-181a is pivotal in unveiling the mechanisms that underlie T cell activation and immune function. YY1 emerges as a central orchestrator in TCR signaling by upregulating miR-181a. This upregulation, in turn, plays a crucial role in mitigating negative feedback loops mediated by miR-181a targets. The intricate interplay between YY1 and miR-181a provides insights into the delicate equilibrium required to finely adjust T cell activation and immune response efficiency. A noteworthy aspect of this review is the examination of the association between miR-181a expression and age-related immune deficiencies. As miR-181a levels diminish in older individuals, the consequences for T cell activation become apparent [11]. These age-related immune deficiencies can be attributed to reduced transcription of YY1, and grasping this connection is fundamental to addressing age-related immune challenges. To emphasize the significance of YY1 in T cell signaling, this review also delves into the consequences of partial YY1 silencing in T cells from young individuals. This approach mirrors the signaling defects observed in older T cells, further under-scoring the pivotal role of YY1 in maintaining T cell responsiveness. This research un-ravels the intricate interplay between YY1 and miR-181a, shedding light on their pivotal roles in TCR signaling and immune response regulation [11]. The decline in miR-181a with age and its connection to reduced YY1 transcription offer valuable insights into the mechanisms underpinning age-related immune deficiencies. Understanding these processes is a significant stride toward devising strategies to enhance T cell activation and address age-related immune challenges.

The effectiveness of protective vaccine responses, reliant on the production of antigen-specific antibodies and the formation of enduring memory T cells, diminishes progressively as individuals age [12]. An essential regulatory component in T cell responses is the dynamic network of microRNAs governing the T cell proteome. The activation-induced increase in miR-21 levels emerges as a central influencer that steers differentiating T cells away from memory T cell development, favoring the generation of inflammatory effector T cells instead. This inclination toward inflammation characterizes T cell responses in older individuals, who exhibit heightened miR-21 expression [12]. In this exploration, we investigate the potential of miR-21 antagonists to counteract this bias and reinstate a more balanced T cell response. By delving into the mechanisms behind miR-21's actions, we uncover its role in targeting negative feedback circuits within multiple signaling pathways. The sustained activity of these pathways in miR-21 high T cells hinders the induction of transcription factor networks crucial for memory cell differentiation. A comprehensive understanding of these intricate mechanisms is vital in our quest to enhance vaccine responses in older individuals. This data suggests that restraining miR-21 upregulation or activity in older individuals could hold the key to bolstering their capacity to mount effective vaccine responses [12]. By mitigating the transcriptome bias toward inflammation and restoring the equilibrium between memory and effector T cells, we can lay the groundwork for more successful vaccination outcomes in the elderly population. The complex interplay between miR-21 and T cell responses, particularly in the context of aging and vaccine effectiveness, underscores the potential of miRNA-based interventions to reinvigorate the immune system's ability to generate protective responses and offer a brighter prospect for public health.

A key finding emphasized in this review revolves around the impact of miR-21 on T cell transcriptomes [13]. Elevated expression of miR-21 during T cell activation directs the differentiation process away from memory T cells and toward inflammatory effector T cells. This shift is a distinctive feature of T cell responses in older individuals, where increased miR-21 expression is observed. Understanding the influence of miR-21 on transcriptomes is crucial for addressing the diminished vaccine responses associated with aging. As the mechanisms governing the actions of miR-21 are unveiled, it becomes apparent that miR-21 targets negative feedback loops within various signaling pathways. The prolonged activation of these pathways in miR-21 high T cells hinders the induction of transcription factor networks essential for memory cell differentiation. By disrupting these negative feedback loops, miR-21 disturbs the delicate balance between memory and effector T cell responses. Our data strongly imply that mitigating miR-21 upregulation or activity in older individuals could hold the key to reinvigorating their capacity to mount effective vaccine responses. Reestablishing the equilibrium between memory and effector T cells is imperative for strengthening the aging immune system and enhancing its responsiveness to vaccination. This research casts light on the potential of miR-21 as a target for augmenting vaccine responses in the elderly [13]. By gaining insights into how miR-21 shapes T cell responses and contributes to age-related immune deficiencies, we can envision innovative strategies that leverage the potential of miRNA-based interventions. This approach offers a promising avenue for revitalizing the aging immune system, ultimately leading to more efficacious vaccine outcomes in older individuals.

A separate study highlights the potential of the demethylating agent 5-Aza-2′-deoxycytidine in promoting increased levels of both the primary transcript and mature miR-142-5p/3p in mesenchymal cells [14]. This observation suggests that DNA methylation plays a role in epigenetically repressing mir-142. The identification of the transcription start site situated within a highly conserved CpG island provides insights into the fundamental/proximal mir-142 promoter and the intricate regulatory mechanisms governing it. Researchers have unveiled a compelling connection between DNA methylation and miR-142-5p/3p expression. In mesenchymal cells with low miR-142-5p/3p levels, both CpG islands were notably methylated, while they remained unmethylated in hematopoietic cells with abundant miRNA expression. Additionally, treatment with 5-Aza-2′-deoxycytidine significantly reduced DNA methylation, resulting in heightened miR-142 expression. This discovery underscores the crucial role of DNA methylation in modulating miRNA levels [14]. The study also demonstrates that the overexpression of miR-142-5p/miR-142-3p can effectively suppress the proliferation of cells with epigenetically silenced endogenous mir-142. This finding holds particular significance, as these miRNAs have been reported to undergo dysregulation in mesenchymal-origin tumors. The functional implications of miR-142-5p/miR-142-3p, coupled with its epigenetic control via DNA methylation, contribute to a deeper under-standing of microRNA regulation and its involvement in various cellular processes. This research unveils the intricate network of epigenetic regulation that governs miR-142 family through DNA methylation, offering valuable insights into the transcriptional control of miRNAs and their functional consequences. Consequently, this study enriches the expanding body of knowledge within the realm of miRNA regulation, emphasizing the intricacy of epigenetic mechanisms in shaping cellular responses.

## 3 Immunity and cardiovascular disease risk factors

### 3.1 Obesity and epicardial adipose tissue

Obesity and diabetes stand out as significant contributors to CVD, warranting focused efforts to unravel the intricacies of CVD development. Adipose tissue (AT), traditionally seen as a fat reservoir, has evolved into a dynamic endocrine organ with widespread implications, exerting a central influence on systemic inflammation and impacting the metabolism, function, and progression of cardiovascular cells. The interaction between the immune system and adipose tissue has led to an enhanced understanding of obesity as an immunometabolic disease. One of the main aspects of this relationship is the direct increase in the volume of epicardial adipose tissue (EAT), which is known to surround the heart and large blood vessels, and its volume directly correlates with the occurrence of coronary heart disease (CHD) [15]. It has been shown that cases of sudden death associated with CHD show increased expression levels of miR-34a-3p/5p in EAT, highlighting the importance of these miRNAs in systemic inflammation caused by impaired fat metabolism. In addition, it is known that miR-34a is involved in suppressing the polarization of anti-inflammatory M2 macrophages, while simultaneously stimulating the activity of pro-inflammatory M1 macrophages and cytokines in visceral AT in vivo [16]. Whole-genome sequencing data from EAT in patients with CHD revealed increased expression of genes associated with antigen presentation, chemokine signaling and systemic inflammation, and decreased expression levels of miR-103-3p, which is responsible for the regulation of Th2 chemokines such as chemokine (CC motif) ligand 13 (CCL13) [17]. Another interesting link involves the presence of EAT around the left atrium and its association with atrial fibrillation (AF). Analysis of miRNA profiles associated with EAT around the left atrium in AF patients reveals the involvement of miR-155-5p and miR-302a-3p, both playing roles in the regulation of interleukin 8 (IL-8), a factor implicated in leukocyte trafficking and activation. The predicted target genes of these miRNAs are associated with cardiac hypertrophy and adipogenesis [18,19]. This intricate interplay involving obesity, inflammation, miRNAs, and cardiovascular disease underscores the multifaceted nature of CVD risk factors and provides valuable insights into potential therapeutic avenues. These findings highlight the complexity of CVD and emphasize the importance of adopting a comprehensive approach to comprehend and address its underlying mechanisms.

### 3.2 Hypertension

The critical involvement of immune cells in the development of hypertension was recognized through pioneering research by Guzik and colleagues back in the mid-2000s, who demonstrated that mice lacking T and B cells, particularly Rag1−/− mice, were particularly protected from hypertension caused by activation of angiotensin II (Ang-II) and deoxycorticosterone acetate. This finding emphasized the key role of T cells as one of the main mediators in the development of hypertension. Notably, reintroduction of T cells, but not B cells, via adoptive transfer was sufficient to restore the hypertensive effects observed in control groups, highlighting the central role of T cells [20].

In one study targeting Ang-II activation-induced hypertension, miR-214-3p, a member of the miR-199/214 cluster, showed a marked eight-fold increase in expression in perivascular tissue in vivo (Ang-II activation-induced hypertension vs. wild-type (WT) model as control). Additionally, miR-214−/− mice undergoing Ang-II infusion dis-played a significant reduction in vascular stiffening, perivascular fibrosis, and hyper-tension, compared to control group. Effects were intricately linked to specific alterations in the T cell transcriptome within miR-214−/− mice, ultimately resulting in reduced T cell activation and diminished infiltration into perivascular adipose tissue. Importantly, elevated plasma levels of miR-214-3p were detected in hypertensive patients, high-lighting the clinical relevance of these findings [21]. In another in vivo study investigating Ang-II-induced hypertension, the deletion of miR-31, a miRNA closely associated with Treg and Th17 cells (miR-31−/−), resulted in a significant reduction in hyper-tension. This reduction was accompanied by diminished cardiac hypertrophy and fibrosis, as well as a decrease in kidney-infiltrating macrophages and an increase in kidney-specific Tregs. Consequently, miR-31−/− mice displayed reduced vascular and renal damage, attributed to the post-transcriptional regulation of protein phosphatase 6C (PPP6C), a direct target of miR-31−/− [22]. These findings underscore the pivotal roles played by T cells and specific miRNAs like miR-214-3p and miR-31 in the development of hypertension and related cardiovascular complications. They not only illuminate the intricate molecular mechanisms underlying hypertension but also offer promising prospects for potential therapeutic targets in its management.

### 3.3 Atherosclerosis

Atherosclerosis is widely acknowledged as an inflammatory and immune-mediated condition, with a central role played by macrophages in the formation of arterial plaques and disease progression. The initial step involves the entrapment of low-density lipoprotein (LDL) beneath the endothelial lining, leading to oxidation and the generation of oxidized LDL (oxLDL). This, in turn, activates ECs, initiating the recruitment and infiltration of monocytes and macrophages. As macro-phages take up oxLDL and internalize apoptotic cell fragments, lipid accumulation within the macrophages results in the formation of foam cells (FCs) [23]. Extensive re-search has focused on understanding the regulatory role of miRNAs in macrophages during the progression and regression of atherosclerosis. As suggested by Xu et al., macrophages-related miR-133b-3p plays a potentially pro-atherogenic role by activating mastermind-like protein 1 (MAML1) expression [24]. This finding aligns with previous research highlighting the significance of NOTCH signaling in leukocyte recruitment and the progression of atherosclerosis. However, further investigation is needed to elucidate the specific mechanisms linking MAML1 release through miR-133b-3p downregulation to NOTCH pathway modulation [[25], [26], [27]]. MiR-17-5p is a noteworthy miRNA with potential as a circulating biomarker for assessing the severity of atherosclerosis [28]. It regulates lipid accumulation and inflammation within macrophages during atherosclerotic processes progression by directly targeting ATP-binding cassette transporter A1 (ABCA1) [29]. ABCA1 is crucial for cellular reverse cholesterol transport and lipid efflux. Downregulation of ABCA1, facilitated by miR-17-5p and other miRNAs like those in the miR-33 family, restricts cholesterol efflux, promoting lipid buildup, and contributing to the development of atherosclerotic plaques [[30], [31], [32]].

Moreover, miR-33-5p is an additional miRNA that has been observed to influence macrophage function, particularly in the context of atherosclerosis. It modulates the expression of peroxisome proliferator-activated receptor-γ coactivator 1-α (PGC1α), a protein that serves as the main regulator of energy metabolism and mitochondrial biogenesis. By diminishing ATP production, miR-33-5p disrupts cholesterol efflux via ABCA1, resulting in the formation of lipid-rich rafts. This amplifies pro-inflammatory extracellular signaling pathways, worsening inflammatory processes [33]. Both miR-17-5p and miR-33-5p play pivotal roles in the intricate mechanisms steering atherosclerosis, and comprehending their functions is crucial for formulating targeted therapeutic approaches for this vascular disease. Adding intricacy to the elaborate molecular mechanisms in atherosclerosis, recent research has identified miR-205-5p as a contributor to the formation of lipid rafts within macrophages [34]. This involvement promotes the development of pro-inflammatory macrophage phenotypes, broadening our understanding of how miRNAs are instrumental in governing lipid transport within macrophages, underscoring their significance in atherosclerosis. In summary, macro-phages, under the regulatory influence of diverse miRNAs, hold a pivotal role in the development of atherosclerosis, overseeing critical processes such as lipid metabolism, inflammation, and the formation of arterial plaques. These insights present promising avenues for potential therapeutic strategies in the context of atherosclerotic diseases [35].

Another crucial aspect in the context of atherosclerosis is impaired efferocytosis, the process through which macrophages clear apoptotic cells and foam cells. This dysfunction is considered a potential contributor to inflammation and the progression of atherosclerosis. In hyperlipidemic Apoe−/− mice, reduced expression of miR-378a-3p in aortic macrophages was linked to the derepression of signal regulatory protein α (SIRPα), which acts as a transmembrane signaling receptor interacting with CD47, known as the "don't eat me" signal [36]. The upregulation of the CD47: SIRPa axis inhibits the clearance of apoptotic cells within plaque lesions, hastening the advancement of atherosclerosis. This study suggests that dysregulated communication between CD47 and SIRPα may also underlie the progression of atherosclerosis in humans. In addition, markedly increased expression levels of extracellular long non-coding RNAs (lncRNAs), such as myocardial infarction associated transcript (MIAT) have been observed in the serum of patients with atherosclerosis, especially those exhibiting symptoms of vulnerable atherosclerotic plaques. This observation implies that MIAT could potentially serve as a valuable biomarker for assessing plaque stability and the severity of atherosclerosis. These findings provide insights into the intricate interplay of molecular factors in atherosclerosis and present potential targets for therapeutic interventions and diagnostic strategies.

A few studies have demonstrated that MIAT acts as a competitive endogenous RNA (ceRNA) for miR-149-5p in macrophages [37]. MIAT possesses binding sequences that complement miR-149-5p, enabling it to sequester miR-149-5p away from CD47, ultimately leading to the derepression of CD47 expression. Such considerations are common concerns in studies aiming to comprehend the endogenous regulators of miRNA activity. However, the concept presented in this work highlights the importance of considering the complex relationships that regulate the functional activity of specific miRNAs within a specific cell type of the immune system such as macrophages [38].

Atherosclerosis is significantly impacted by various types of white blood cells, with T cells, particularly those of the CD4^+^ lineage, playing a substantial role in its development. Abnormal expression of miR-142-3p may influence the recruitment of CD4^+^ T cells into vascular walls, affecting cytoskeleton dynamics [39]. In atherosclerotic plaques, CD4^+^ type 1 T helper (Th1) cell responses, regulated by the master transcriptional regulators TBX21 (T-BET) and eomesodermin (EOMES), represent the predominant T cell subset. These Th1 responses contribute to the progression and severity of atherosclerosis [[40], [41], [42]]. The miR-29 family, including miR-29a-3p and miR-29b-3p, functions to restrict the expression of these target genes and in T cells, potentially mitigating atherosclerosis and enhancing plaque stability by directly inhibiting Th1 development [43]. However, evidence from studies using locked nucleic acids (LNAs) to broadly inhibit miR-29 members suggests that in specific cell types, these miRNAs may indeed contribute to disease progression. Nevertheless, this specific study did not explore the impact of LNA-miR-29 on the development of Th1 responses. Therefore, further investigations are required to comprehensively understand the contribution of miR-29 family members originating from T cells to the promotion of Th1 development within atherosclerotic plaques, in both human and murine diseases. Such research is vital for guiding potential therapeutic strategies.

### 3.4 Diabetes

Diabetes mellitus, particularly Type 1 (T1D) and Type 2 diabetes (T2D), is characterized by metabolic dysregulation and chronic hyperglycemia, which collectively induce immune responses that elevate cardiovascular disease (CVD) risk. This chronic hyperglycemic state activates inflammatory pathways and oxidative stress, which disrupts immune homeostasis and contribute to the pathogenesis of diabetic complications, including CVD [[44], [45], [46]]. In both T1D and T2D, immune dysregulation is a significant contributor to vascular inflammation and related complications. Hyperglycemia promotes an environment of increased pro-inflammatory cytokine production, such as TNF-α, IL-6, and IL-1β, mediated through pathways like NF-κB. This leads to macrophage activation, promoting the M1 inflammatory phenotype over the reparative M2 phenotype, resulting in chronic inflammation within vascular tissues [44]. Additionally, in T1D, autoimmune destruction of pancreatic β-cells is driven by autoreactive T cells, which are typically regulated by Tregs. However, Treg function and stability are often compromised in diabetic conditions, leading to persistent autoimmunity and immune activation [45].

Several miRNAs act as critical regulators of immune responses in diabetes by modulating macrophage activation, T cell responses, and cytokine signaling. miR-146a: Known for its anti-inflammatory properties, miR-146a suppresses NF-κB signaling by targeting IRAK1 and TRAF6. In diabetes, miR-146a dysregulation enhances inflammatory responses, increasing susceptibility to CVD. Upregulation of miR-146a has been shown to reduce inflammation and slow disease progression in diabetic models by reducing M1 macrophage activation and promoting anti-inflammatory pathways [43,44]. MiR-155 - this pro-inflammatory miRNA is often elevated in diabetic conditions, promoting macrophage M1 polarization and enhancing cytokine production. miR-155 overexpression exacerbates inflammation in the vascular endothelium, contributing to plaque formation in atherosclerosis and increasing the risk of CVD in diabetic patients [44]. miR-21 is upregulated in various diabetic complications and contributes to fibrosis and vascular inflammation. In diabetes-related CVD, miR-21 enhances fibroblast activation and macrophage infiltration, thus promoting adverse cardiac remodeling. Targeting miR-21 could reduce inflammation and fibrosis, improving cardiovascular outcomes in diabetes [44].

Targeting these miRNAs presents a promising strategy for managing diabetes-related immune dysregulation and reducing CVD risk. By modulating miRNA expression, therapies could attenuate inflammatory responses, restore immune balance, and potentially reduce vascular complications. Therapeutic modulation of miR-146a, miR-155, and miR-21 could alleviate the progression of both diabetes and associated cardiovascular diseases, presenting a dual approach to managing these interconnected health challenges [45].

## 4 The regulation of immune cells by miRNAs in the context of CVDs and its related complications

### 4.1 Myocardial infarction

Monocytes and macrophages play crucial roles in initiating inflammation and inducing cardiomyocyte apoptosis during the initial inflammatory response following a myocardial infarction (MI) [47]. Their involvement extends into the later phases of left ventricular remodeling and enlargement, ultimately leading to heart failure. MiR-21-5p emerges as a crucial intrinsic mediator that suppresses the polarization of M1 macrophages in the post-MI cardiac environment. The expression of miR-21-5p rapidly in-creases within the infarcted regions of cardiac tissue in vivo. Remarkably, in mice lacking miR-21 (miR-21−/−) and subjected to MI, the outcomes are characterized by higher mortality rates, larger infarct sizes, and increased scarring. MiR-21-5p, expressed by macrophages, directly restrains the inflammation and cytokine synthesis by targeting Kelch repeat and BTB (POZ) domain-containing 7 (KBTBD7), a transcriptional activator involved in mitogen-activated protein kinase signaling (MAPK). In the absence of miR-21-5p, the overexpression of KBTBD7 enhances macrophage activation by promoting the p38 and nuclear factor kappa-light-chain-enhancer of activated B cells (NF-kB) pathways [48].

To strengthen the regulatory processes of miR-21, a promising therapeutic strategy involves the intravenous nanoparticles (NPs) loaded with miR-21-5p mimics, specifically targeted to cardiac macrophages in the early stages of MI induction [49]. This approach aims to diminish pro-inflammatory responses and promote the development of reparative, wound-healing macrophage phenotypes, potentially alleviating pathological remodeling. Another encouraging avenue for therapeutic intervention involves modifying the development of M1 macrophages in post-MI by targeting miR-375-3p in macrophages [50]. This approach activates 3-phosphoinositide-dependent protein kinase 1 and reduces the expression of pro-inflammatory cytokines. Additionally, the expression of NADPH oxidase 2 (NOX2), an enzyme producing superoxide, increases in cardiomyocytes and macrophages post-MI [[51], [52], [53]]. NOX2 promotes the inflammatory polarization of macrophages and the expression of cytokines, associated with adverse cardiac remodeling post-MI. In both human and animal macrophages, miR-106b-5p, miR-204-5p, miR-148b-3p, and have demonstrated the ability to suppress NOX2. Enhancing these miRNAs through targeted delivery using pH-sensitive polyketal nanoparticles to macrophages significantly enhances post-MI outcomes, leading to notable improvements in infarct size and cardiac function. This approach represents another potentially effective therapeutic strategy for managing MI [54].

### 4.2 Heart failure

Heart failure (HF) is frequently marked by persistent inflammation, characterized by increased levels of pro-inflammatory cytokines such as interleukin-6 (IL-6), interleukin-1b (IL-1b), and tumor necrosis factor alpha (TNF-a) in the blood of HF patients. Analysis of the CANTOS phase III clinical trial data reveals that canakinumab (Ilaris), an anti-IL-1b monoclonal antibody, demonstrated a reduction in HF-related hospitalizations and mortality in post-MI patients [55]. However, phase III trials aiming to target HF through anti-inflammatory interventions have generated fewer promising results, indicating that inflammatory responses may serve as a primary driver of HF in specific subsets of patients [56]. In individuals with dilated cardiomyopathy (DCM), excessive activation and proliferation of CD4^+^ T cells have been linked to decreased expression of miR-451a-5p and increased expression of Myc [57]. This underscores the importance of T cell responses and their regulation by miRNAs in the progression of HF. Moreover, the decline in immune function associated with aging, known as immunosenescence, is closely linked to HF development. Specific miRNAs associated with the aging process, such as members of the miR-181 family and miR-34a-5p, may contribute to these effects by modulating lymphocyte function [58]. Targeting these molecules holds the potential to alleviate the progression of aging-related HF. In summary, inflammation is intricately linked to chronic HF, with its impact varying among different patient groups. A comprehensive understanding of the complex interplay between immune responses, aging, and miRNA regulation could provide valuable insights into more effective therapeutic approaches for managing HF, particularly considering the changes in the immune system associated with aging (Table 1).

**Table 1:** Key microRNA (miRNAs), their immune cell origin, their function in immune regulation, and their association with cardiovascular diseases (CVDs).

miRNA	Immune Cell Origin	Function in Immune Regulation	Association with CVD
miR-146a	Macrophages, T cells	Anti-inflammatory, inhibits NF-κB pathway	Reduces vascular inflammation, protective in atherosclerosis
miR-155	Macrophages, T cells	Pro-inflammatory, promotes M1 polarization	Increases inflammation, contributes to plaque instability
miR-21	Macrophages	Pro-fibrotic, promotes fibroblast activation	Promotes fibrosis and vascular inflammation, linked to adverse remodeling
miR-223	Monocytes, Neutrophils	Regulates inflammation, affects M1/M2 balance	Regulates macrophage polarization, associated with plaque stability
miR-126	Endothelial cells, Platelets	Anti-inflammatory, inhibits leukocyte adhesion	Inhibits leukocyte adhesion to endothelium, protective in atherosclerosis
miR-34a	Macrophages, T cells	Induces apoptosis, involved in cell cycle arrest	Involved in atherosclerosis, promotes endothelial cell apoptosis
miR-125b	Dendritic cells, T cells	Promotes macrophage polarization towards M2	Supports tissue repair, beneficial in myocardial infarction
miR-126-5p	Endothelial cells, Monocytes	Anti-inflammatory, reduces endothelial dysfunction	Protects against vascular inflammation, reduces plaque formation
miR-92a	Macrophages, Platelets	Inhibits inflammatory cytokine release, anti-apoptotic	Limits inflammation, beneficial in vascular injury recovery
miR-27b	Neutrophils, Endothelial cells	Regulates inflammatory signaling, limits cell adhesion	Decreases endothelial adhesion molecule expression, protective against thrombosis

## 5 The therapeutic potential of miRNA-regulated pathways in the context of CVD for Tregs immunotherapy

Considerable research efforts have been dedicated to exploring Tregs as potential targets for treating medical conditions associated with immunological dysfunctions [[59], [60], [61]]. These investigations encompass a range of health issues, including, autoimmune disorders like diabetes, graft-versus-host disease (GvHD), Crohn's disease, and solid organ transplantation, where establishing enduring tolerance to transplanted organs is a fundamental clinical goal. As the understanding of the di-verse ways in which Tregs provide protection to the cardiovascular system in CVD expands, and with a growing body of evidence implicating imbalances in Tregs numbers and their suppressive functions in CVD pathogenesis, there is a rising interest in immunotherapy involving exogenously introduced Tregs or targeted treatments specific to Tregs. This avenue holds promise as a novel and innovative therapeutic approach. The advancing knowledge in this field has the potential to pave the way for new interventions that harness the capabilities of Tregs to modulate immune responses and alleviate diseases rooted in immunological dysregulation [[62], [63], [64]]. This research is anticipated to shape the future of immunotherapy and contribute to more effective treatments for a wide range of conditions related to immune system dysfunction [[65], [66], [67]].

In recent years, there has been a significant emphasis on investigating the roles of miRNAs concerning Tregs and their functions. Numerous research studies have underscored the crucial impact of specific miRNAs on Tregs biology, encompassing their development, differentiation, and suppressive activities. This body of research has unveiled the intricate landscape of miRNA-mediated regulation in Tregs. A key facet of Tregs function involves the production of cyclic adenosine monophosphate (cAMP), a central player in suppressing effector T cells. Notably, miR-142 isoforms have been identified as pivotal regulators of cAMP production within Tregs. For instance, miR-142-3p is downregulated in Tregs to ensure the expression of ADCY9, a critical factor in cAMP production that contributes to the suppressive capabilities of Tregs. Additionally, miR-142-5p plays a crucial role in suppressing cAMP hydrolase, phosphodiesterase 3B (PDE3B). Dysregulation of miR-142-5p in regulating PDE3B leads to a breakdown of peripheral immune tolerance, resulting in widespread immune cell activation and extensive tissue damage in murine models. Furthermore, miR-142-3p has emerged as a significant regulator of TGF-beta-mediated Tregs development by influencing transforming growth factor beta receptor 1 (TGFBR1) [68]. Deleting the miR-142 locus has been shown to promote enduring tolerance to cardiac allografts, underscoring the indispensable role of miR-142 isoforms in shaping the development and functionality of Tregs. This intricate understanding of miR-142's regulatory role provides valuable insights into the potential for innovative interventions in immune modulation. It highlights the significance of miRNAs in molding immune responses and offers opportunities for developing more effective treatments for conditions rooted in immunological dysregulation [69]. The evolving comprehension of miRNAs in Tregs biology holds great promise for the future of immunotherapy, presenting the potential to fine-tune immune responses and address a broad spectrum of diseases associated with immune system dysfunction. This research has the potential to revolutionize the field of immunotherapy, ultimately improving the lives of individuals with various immunological disorders [70].

However, there exists a significant knowledge gap in comprehending how these discoveries and the broader implications of miRNA-regulated Tregs are pertinent to CVD. Despite substantial evidence supporting the protective roles of Tregs in CVD, there is a noticeable dearth of research characterizing the potential of Tregs therapy in major CVD conditions, such as atherosclerosis [71]. The notion of using miRNA mimics and synthetic miRNA inhibitors (antagomiRs) as therapeutic agents has garnered considerable attention [72,73]. Early-stage candidates designed for various conditions, including hepatitis C infection (e.g., Miravirsen), cancer (e.g., Cobomarsen (MRG106)), and ischemia (e.g., MRG-110), have shown promise in phase I and II clinical trials. Additionally, pre-clinical data from experiments involving antagomiR-treated Tregs in humanized mouse models of GvHD suggest that miRNA modulation of Tregs holds promise for enhancing Tregs stability and their suppressive activity in the context of adoptive cell transfer immunotherapy ex vivo [74].

The potential of targeted miRNA manipulation of Tregs offers a promising approach to overcoming some of the expected limitations of adoptive transfer Tregs immunotherapy (Table 2) [[75], [76], [77], [78], [79], [80], [81], [82], [83], [84], [85], [86], [87], [88], [89]]. This includes stabilizing specific regulatory phenotypes and improving control over their migration to desired sites, which holds potential benefits for both CVD and other medical conditions. These insights raise the possibility that correcting dysfunctional Tregs in specific forms of CVD might be therapeutically achieved by targeting aberrantly expressed miRNAs within ex vivo expanded populations of the patient's cells. This approach becomes particularly important when considering that while interventions targeting resident or infiltrating cell populations in the cardiovascular system may be convincingly effective in treating CVD, where untargeted systemic immune suppression and inflammatory responses can potentially lead to unintended general or opportunistic infections and other adverse effects.

**Table 2:** A concise overview of microRNAs (miRNAs) and their recognized targets in the control of regulatory T cells (Tregs) function.

MiRNA	Regulation	Targets	Biological effects	Reference
miR-4281-3p	Up	FOXP3	The positioning and interaction of miR-4281-3p within the cell nucleus with the TATA-box in the FOXP3 promoter led to an increased expression of FOXP3. This, in turn, contributed to the improvement of Tregs differentiation, persistence, and overall functionality.	[75]
miR-142-5p	Down	PDE3B	The absence or significant reduction of miR-142 expression in Tregs led to impaired peripheral tolerance, resulting in widespread systemic autoimmune process and inflammation. This is due to the reduced level of cAMP expression and inactivation of Tregs.	[76]
miR-142-3p	Down	TGFBR1, TET2 and KDM6A	Inactivation of T cell-related miR-142 prevented the rejection of MHC-mismatched heart and skin allografts by increasing the intragraft Treg population and by increasing TGFBR1 expression. In the context of pancreatic islet autoimmunity, reducing miR-142-3p levels restored Treg induction and stability during the disease. Decreased expression of miR-142-3p led to increased expression of FOXP3 and enhanced suppressive abilities of Tregs by increasing the expression levels of KDM6A and BCL-2 through demethylation of H3K27me3.	[[77], [78], [79]]
miR-155-5p	Down	SOCS1	FOXP3 promotes the expression of miR-155-5p in Tregs. Genetically lacking miR-155 impairs STAT5 signaling, resulting in a reduction in Treg numbers and overall balance.	[80]
miR-15a-5p/16-5p	Up	FOXP3	Forced overexpression suppresses FOXP3 and reduces the suppressive capabilities of the cells.	[81]
miR-24-3p	Down	FOXP3	The natural expression of miR-24-3p in Tregs is typically low. However, artificially increasing its expression leads to a reduction in FOXP3 expression.	[82]
miR-125a-5p	Down	STAT3, IFNG and IL-13	The absence of miR-125a-5p results in a reduction of Tregs and promotes inflammatory responses, worsening conditions such as colitis and EAE. MiR-125a is involved in preserving the equilibrium and stability of Tregs.	[83]
miR-146a-5p	Down	STAT1	The lack of miR-146a-5p in Tregs interferes with tolerogenic processes and promotes Th1 responses.	[84]
miR-181a/b-5p	Down	CTLA4	The deficiency of miR-181a/b-5p inhibits the formation of thymic Tregs while augmenting the suppressive capabilities of peripheral Tregs.	[85]
miR-146b-5p	Down	TRAF6	Employing an antagomir to downregulate miR-146b-5p improves the efficacy of thymic Tregs and enhances their inhibitory impact on graft-versus-host disease (GvHD).	[86]
miR-202-5p	Up	MATN2	In the context of allergic rhinitis, elevated levels of miR-202-5p impede both the formation and the effectiveness of Tregs.	[87]
miR-340-5p	Down	IL-4	In allergic rhinitis, the heightened expression of miR-202-5p impedes both the formation and the efficacy of Tregs.	[88]
miR-1224-5p	Down	FOXP3	AhR signaling alleviates systemic inflammation triggered by pertussis toxin by suppressing miR-1224-5p, elevating FOXP3 expression, and promoting the maturation of Tregs.	[89]

TheranomiRNAs, or therapeutic miRNAs with diagnostic and treatment capabilities, represent a promising approach in the management of CVDs, especially for conditions involving immune dysregulation [5]. These miRNAs have dual potential: they can act as biomarkers for early detection and as therapeutic agents to modulate underlying disease mechanisms, providing a more targeted and personalized therapeutic strategy. In CVD, immune dysregulation often results in chronic inflammation and vascular remodeling, contributing to the progression of atherosclerosis, heart failure, and other cardiovascular complications. Certain theranomiRNAs can target specific components of immune and inflammatory pathways. Known for its anti-inflammatory properties, miR-146a inhibits the NF-κB pathway, reducing the expression of pro-inflammatory cytokines like IL-6 and TNF-α. Administering miR-146a mimics could mitigate inflammation in atherosclerotic plaques, potentially stabilizing them and lowering the risk of plaque rupture. This action could be especially valuable in patients at high risk of heart attack or stroke While often elevated in fibrotic and inflammatory conditions, targeted inhibition of miR-21 has shown potential in reducing fibrosis and adverse remodeling. In CVD management, anti-miR-21 therapies could help prevent excessive fibroblast activation and collagen deposition, protecting against heart failure progression by addressing fibrosis at its molecular root. Given its role in promoting macrophage M1 polarization and inflammation, miR-155 inhibition is being explored to reduce inflammatory responses in vascular tissues. By downregulating miR-155, it may be possible to shift macrophage activity from a pro-inflammatory to a reparative M2 phenotype, which could help stabilize atherosclerotic plaques and improve vascular health. miR-223 - this miRNA modulates the balance between pro-inflammatory M1 and anti-inflammatory M2 macrophages. Enhancing miR-223 expression could promote anti-inflammatory responses, contributing to plaque stability and reducing inflammation. As a theranomiR, miR-223 also shows promise as a biomarker for CVD progression, providing insight into immune activity within cardiovascular tissues [5].

The targeted use of theranomiRNAs provides several advantages over traditional therapies. First, miRNAs offer a high degree of specificity, as they can be designed to target mRNA sequences associated with CVD pathology, thereby reducing off-target effects. Second, theranomiRNAs enable a non-invasive approach to CVD management. Because they can circulate in the bloodstream, they can serve as biomarkers detectable through blood tests, allowing for regular monitoring of disease progression and treatment efficacy. Lastly, theranomiRNAs allow for the modulation of immune responses at multiple stages of the inflammatory cascade, providing a more comprehensive approach to disease modification. For theranomiRNAs to reach their full potential in clinical practice, advances in delivery mechanisms are critical. Nanoparticle-based carriers and viral vectors are under investigation to improve miRNA stability and targeting precision. Additionally, the development of anti-miR oligonucleotides (AMOs) for inhibiting specific miRNAs opens new avenues for managing miRNA-mediated immune dysregulation. TheranomiRNAs hold significant promise for CVD therapy by addressing immune dysregulation at its molecular roots. By modulating key pathways in inflammation and immune cell activation, these miRNAs could reduce disease progression, improve patient outcomes, and herald a new era in personalized cardiovascular medicine.

## 6 Perspectives

Investigating the intricate regulation of immune responses by miRNAs is a rapidly expanding area of research. A profound understanding of these complex regulatory pathways and their specific roles in various immune cells is crucial for unlocking their potential for therapy. In the realm of CVD, it is imperative to explore how miRNAs govern immune responses within the unique context of each specific disease, rather than making broad assumptions. While certain well-established immuno-miRs, like miR-155-5p, have garnered significant attention due to their documented dysregulation in CVD pathogenesis, there are other immuno-miRs that remain relatively unexplored in the regulation of the immune system in CVD [[90], [91], [92]]. A notable example is miR-142, which wields significant influence over immune system development and cellular signaling pathways. Additionally, changes in its circulating levels have been observed in CVD, even though direct research on its involvement in this context is limited.

Building on the significance of in silico analysis, these tools allow for a comprehensive exploration of miRNA interactions, assisting researchers in screening vast numbers of potential miRNAs efficiently. By applying bioinformatic tools to assess miRNA-gene interactions, scientists can predict which miRNAs are most likely involved in specific pathological processes, as demonstrated in the reviewed article. For example, the study highlights miRNAs like miR-133a-3p, miR-21-5p, and miR-499a-5p for their relevance in CVDs. By utilizing databases and algorithms, researchers can generate hypotheses about the miRNAs involved in disease mechanisms, thus prioritizing candidates for experimental studies, which reduces costs and focuses resources on the most promising miRNA biomarkers [5]. The integration of artificial intelligence (AI) in miRNA research is advancing rapidly, and its potential in predicting miRNA functions and interactions adds significant value to CVD research. AI tools can analyze extensive biological datasets, pinpointing intricate miRNA-mRNA regulatory networks with remarkable speed and accuracy. Machine learning algorithms, for instance, can identify patterns in gene expression that may not be evident through traditional methods, enabling AI to propose novel miRNA targets and pathways specific to diseases like atherosclerosis, myocardial infarction, and hypertension. AI-driven approaches have shown promise in the concept of theranoMiRNAs—miRNAs that hold diagnostic as well as therapeutic potential. The recommended article explores how computational models can identify miRNAs, such as miR-21 and miR-126, which could serve as theranoMiRNAs in CVDs due to their role in heart damage and fibrosis. These miRNAs are increasingly studied not only for their diagnostic utility but also as therapeutic targets, providing a potential dual benefit for managing CVDs. Furthermore, AI's predictive capabilities extend to miRNA-based therapeutic design. By simulating miRNA effects on gene networks, AI models can assist in designing miRNA mimics or inhibitors that modulate disease pathways. In cardiovascular medicine, where conditions like heart failure and myocardial infarction involve complex, multi-gene regulation, AI can predict the efficacy and safety profiles of miRNA therapies, paving the way for personalized treatments [5]. This dual approach—using in silico screening to prioritize miRNAs for further studies and AI to design targeted miRNA therapies—could revolutionize how researchers approach CVDs, offering a pathway to precise, personalized miRNA-based interventions.

As highlighted in this review, the involvement of macrophages and CD4^+^ T cell responses is pivotal in the development and progression of CVD. Targeting miRNAs and the signaling networks they govern in these cell types, especially those exerting far-reaching influence over various aspects of immune cell biology, holds promise for immunotherapeutic applications. However, determining the most effective and safe approach to target these molecules, with a focus on specific cell types, remains an open question. The use of modified synthetic antisense oligonucleotides may introduce off-target effects, especially when administered at higher therapeutic doses in vivo. Additionally, these effects may persist over time due to the enhanced stability of such molecules. Innovative packaging technologies, such as modified polymeric NPs, might offer solutions to some of these challenges, facilitating more controlled delivery and release of therapeutic agents into target cells [93].

Alternatively, an alternative strategy involves immunotherapy using miRNA-modified Tregs to suppress dysregulated inflammation and immune responses underlying CVD pathogenesis. This approach requires further exploration and substantial research efforts. Recent breakthroughs in single-cell sequencing and analytical technologies have provided an unprecedented level of insight into rare cardiac immune cell populations, historically challenging to investigate. For instance, it has recently been discovered that a population of tissue-resident innate immune cells, known as group 2-committed innate lymphoid cells (ILC2s), exists in the heart tissue of both mice and humans [[94], [95], [96], [97]]. These cells may exert significant influence in CVD, including conditions like myocarditis, cardiac ischemia, and pericarditis. Furthermore, populations observed in para-aortic adipose tissue and lymph nodes may contribute significantly to the progression of atherosclerosis. However, much of this research has been conducted in mice, and the relevance of these findings to human CVD is yet to be definitively established [[98], [99], [100]]. Subsequent studies should aim to uncover how microRNAs can control function among immune cell populations in both healthy and disease states. This research has the potential to reveal exciting new therapeutic avenues for individuals grappling with CVD.

## 7 Conclusion

This review highlights the multifaceted roles of immune-derived miRNAs in the pathogenesis and progression of cardiovascular diseases (CVDs). Immune cells, such as macrophages, T cells, and neutrophils, contribute to chronic inflammation and tissue remodeling, both key processes in CVD. Specific miRNAs—referred to as “immuno-miRs”—regulate these immune responses by modulating cytokine production, macrophage polarization, and inflammatory signaling pathways. Key miRNAs, including miR-146a, miR-155, miR-21, and miR-223, serve as molecular regulators, influencing both the inflammatory environment and immune cell function within cardiovascular tissues. These immune-derived miRNAs also show significant promise as theranomiRs, a class of miRNAs with diagnostic and therapeutic potential. For example, miR-146a and miR-223 have anti-inflammatory effects that could stabilize atherosclerotic plaques, while miR-21 inhibition may prevent fibrosis and adverse remodeling in heart tissue. These theranomiRs could serve as non-invasive biomarkers, facilitating early detection and monitoring of CVD progression, and as therapeutic agents targeting specific pathways involved in immune dysregulation. The clinical translation of theranomiRNAs holds potential for personalized CVD management by targeting disease mechanisms at the molecular level. Continued research into delivery mechanisms, such as nanoparticle and viral vector systems, will be crucial to realize the full therapeutic potential of immune-derived miRNAs. In conclusion, immune-derived miRNAs represent both a diagnostic and therapeutic frontier in cardiovascular disease, offering hope for more effective and targeted interventions in the future of cardiovascular medicine.

## CRediT authorship contribution statement

**Albert Sufianov:** Supervision, Project administration, Conceptualization. **Murad Agaverdiev:** Writing – review & editing, Writing – original draft. **Andrey Mashkin:** Formal analysis, Data curation, Conceptualization. **Tatiana Ilyasova:** Visualization, Validation, Resources.

## Patient consent for publication

Not applicable.

## Ethics approval and consent to participate

Not applicable.

## Funding

This work was supported by the Bashkir State Medical University Strategic Academic Leadership Program (PRIORITY-2030)

## Conflicts of interest

All authors declare that there are no competing interests.
